# High-throughput Toroidal Grating Beamline for Photoelectron Spectroscopy at CAMD

**DOI:** 10.1088/1742-6596/493/1/012024

**Published:** 2014

**Authors:** O Kizilkaya, R W Jiles, M C Patterson, C A Thibodeaux, E D Poliakoff, P T Sprunger, R L Kurtz, E Morikawa

**Affiliations:** 1 Center for Advanced Microstructures and Devices, Louisiana State University, Baton Rouge, LA 70806, USA; 2 Department of Physics and Astronomy, Louisiana State University, Baton Rouge, LA 70803, USA; 3 Department of Chemistry, Louisiana State University, Baton Rouge, LA 70803, USA

## Abstract

A 5 meter toroidal grating (5m-TGM) beamline has been commissioned to deliver 28 mrad of bending magnet radiation to an ultrahigh vacuum endstation chamber to facilitate angle resolved photoelectron spectroscopy. The 5m-TGM beamline is equipped with Au-coated gratings with 300, 600 and 1200 lines/mm providing monochromatized synchrotron radiation in the energy ranges 25-70 eV, 50–120 eV and 100–240 eV, respectively. The beamline delivers excellent flux (~10^14^-10^17^ photons/sec/100mA) and a combined energy resolution of 189 meV for the beamline (at 1.0 mm slit opening) and HA-50 hemispherical analyzer was obtained at the Fermi level of polycrystalline gold crystal. Our preliminary photoelectron spectroscopy results of phenol adsorption on TiO_2_ (110) surface reveals the metal ion (Ti) oxidation.

## 1. Introduction

The 6 meter toroidal-grating monochromator (6m-TGM) beamline at the Center for Advanced Microstructures and Devices (CAMD) at Louisiana State University was decommissioned in 2008. The 5m-TGM beamline, formally operated at the Electron Stretcher Accelerator (ELSA) Synchrotron Facility, Bonn University, Germany, was relocated to CAMD in 2010 to be installed on the 1.5 GeV CAMD electron storage ring and the same bending magnet port used for the 6m-TGM at CAMD. The beamline commissioning was completed in 2011 and an ultrahigh vacuum (UHV) endstation equipped with a HA-50 electron analyzer was commissioned and attached to the 5m-TGM beamline to facilitate vacuum ultraviolet angle-resolved photoelectron spectroscopy. In this report we present the 5m-TGM beamline performance and the preliminary ultraviolet photoelectron spectroscopy results of phenol adsorption on TiO_2_ (110) acquired from this beamline.

## 2. 5m-TGM Beamline

**Figure 1:**
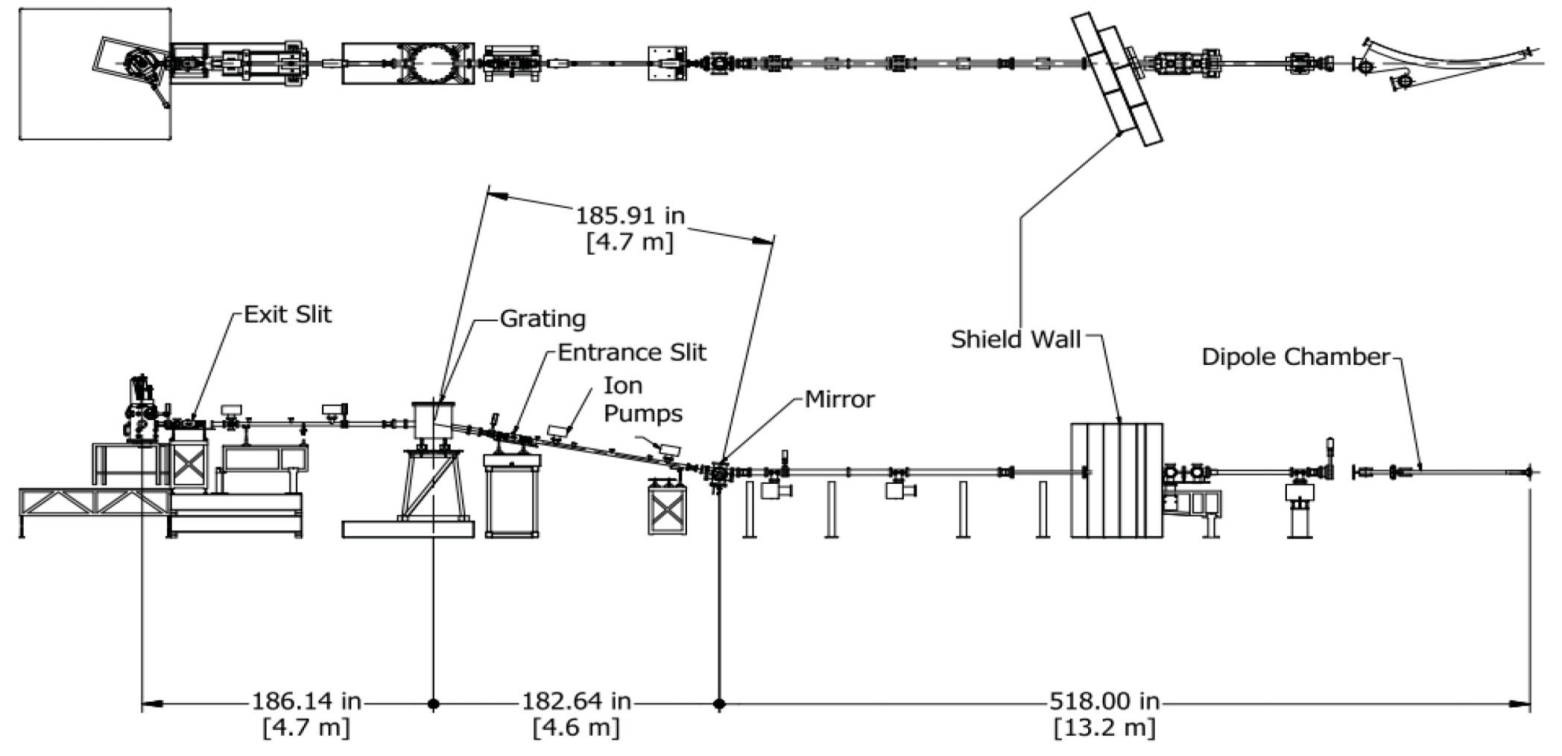
Layout of 5m-TGM beamline at CAMD

The 5m-TGM beamline was utilized for x-ray absorption experiments at the Bonn electron stretcher and accelerator ELSA, operated at 2.3 GeV in the storage-ring mode with an average current of 30 mA [1]. Side and top views of the 5m-TGM beamline configuration at CAMD are shown in figure 1. The first focusing mirror is attached to a vacuum chamber outside of the shield wall. The focusing mirror, which has a pitch adjustment, is the first optical element on the beamline and is a water-cooled ellipsoid. Reflected synchrotron light from the focusing mirror with an incidence angle of 84.5° is focused on the entrance slit. The synchrotron radiation is monochromatized using the toroidal diffraction gratings. The monochromator chamber is equipped with ion-etched, Au-coated gratings with 300, 600, and 1200 lines/mm providing highly resolved monochromatized synchrotron radiation in the energy ranges of 25-70, 50–120 eV, 100–240 eV, respectively. All three toroidal gratings are placed on a cradle, which can be moved horizontally with an extended arm operated with a motor outside of the grating chamber, allowing easy grating exchange. The 5m-TGM beamline does not have a refocusing mirror after the exit slit, and this contributes to the high-throughput nature of the beamline. The monochromatic light from the grating is parallel to the floor and produces a beam-spot size of 5 mm × 2 mm at the sample position with a 1mm vertical and 10 mm horizontal openings on the entrance and exit slits.

The attached ultrahigh vacuum (2×10^−10^ Torr) endstation is primarily used to conduct angle-resolved photoelectron spectroscopy (ARPES) experiments on a variety of materials. The chamber is equipped with a sample load-lock, ion sputtering, and numerous evaporation sources. The sample manipulator allows for full rotation (2-axis) and translation (x-y-z), and facilitates sample temperatures between 90-1600 K. In the case of ARPES data acquisition, photoelectrons emitted from a sample in the endstation are collected with a VSW HA-50 hemispherical electron analyzer, which rotates along 2-axes. This allows for full Brillouin mapping with differing polarizations. The voltages applied to the electrostatic lenses of the analyzer are controlled by a VSW-HAC 300 power supply. The VSW-HAC 300 controller floats over a Valhalla 2701C programmable DC voltage supply controlled by a GPIB interface to set the kinetic energy range of a scan. The charge-signal pulse from a channeltron is capacitively coupled into a voltage signal pulse with an electronic circuit placed outside the vacuum chamber. The pulses are amplified and collected by a timer/counter. A normalization current, proportional to the incoming flux, is also available. A data acquisition program written in LabVIEW program at CAMD records the counts and plots the photoelectron spectrum. The LabVIEW program also controls the grating rotation and its move to an intended photon energy position.

**Figure 2:**
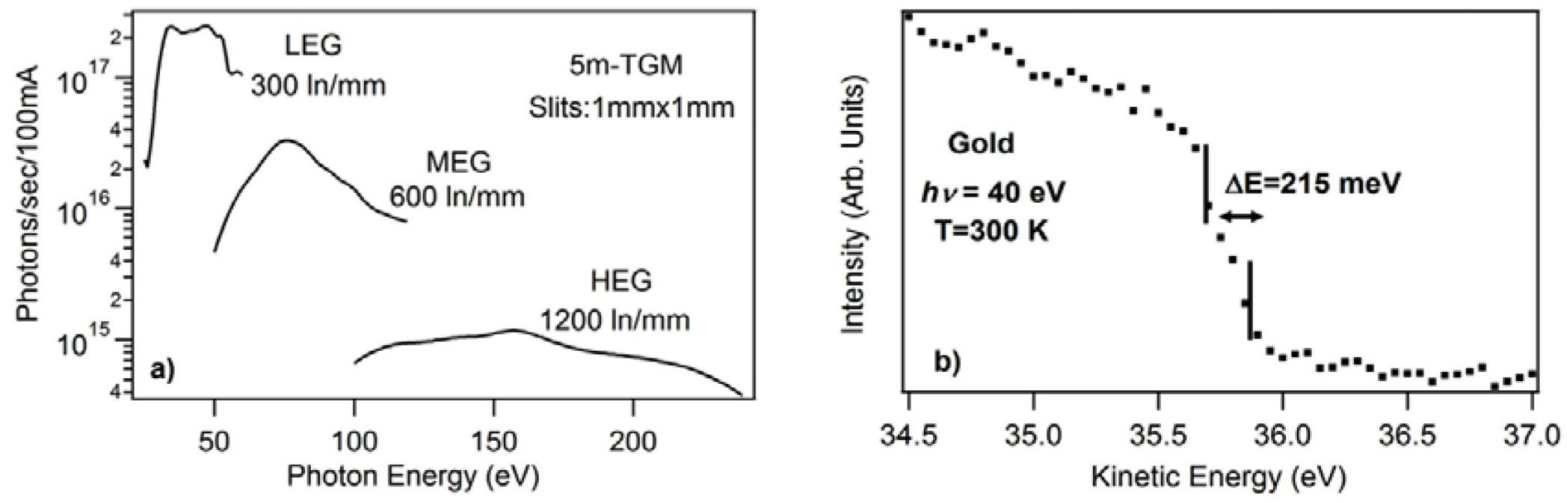
a) Photon flux of LEG, MEG and HEG at the sample position using a GaAsP diode. b) Full width half maximum of the Fermi edge of polycrystalline gold.

Photon flux delivered by each grating was measured with a GaAsP diode placed at the sample position. Absolute quantum efficiency of the diode was used in the photon flux calculations shown in figure 2a. The low energy grating delivers a high photon flux ~ 10^17^ photons/sec/100mA and high energy grating provides a moderate flux of 10^14^-10^15^ photons/sec/100mA. In order to determine the combined beamline and analyzer resolution, a Fermi edge of a clean polycrystalline gold crystal was measured at 40 eV photon energy. The full width half maximum of the Fermi edge, 215 meV (see figure 2b), demonstrates a moderate resolution. By correcting for temperature broadening (~4kT=103 meV at 300K), the combined resolution of 189 meV is obtained with a 1mm entrance and exit slit setting of the beamline. It should be added that substantially better combined resolution will be achieved with decreased slit widths.

## 3. Phenol adsorption on TiO_2_(110)

Recent studies demonstrated that airborne fine and ultrafine particles are mostly generated in combustion sources and these particles cause cardiopulmonary disease and even may trigger cancer [2]. These studies also indicated that airborne particles also contain environmentally persistent free radicals (EPFRs) generated in combustion reactions. The Dellinger group reported that their electron paramagnetic resonance (EPR) experiment demonstrated the formation of environmentally persistent free radicals on metal oxide nanoparticles [3]. Precursors such as phenol initially physisorb on Cu(II)O nanoparticles supported on silica and chemisorption takes place via H_2_O elimination through an electron transfer mechanism from phenol to metal oxide resulting in the reduction of metal ion (Cu(I)O).

Electron energy loss spectroscopy (EELS) and EPR spectroscopy experiments were conducted by our group to unravel the mechanisms of EPFR formation from phenol precursors on the surface of well-characterized metal oxides TiO_2_ (110) and ultrathin films of Al_2_O_3_ grown on NiAl (110) [4]. Neither of the techniques decisively determined the metal oxide reduction after radical formation. On the other hand, EELS data from phenol adsorbed on TiO_2_ indicated that phenol adsorbs the commonly known defect sites. This observation is proposed as a possible mechanism for the oxidation of the metal oxide substrate.

To elucidate the electronic structure and the interaction of phenol with TiO_2_ (110), we performed X-ray and vacuum ultraviolet (VUV) photoelectron spectroscopy measurements. X-ray data was collected in an UHV chamber equipped with an Omicron XM1000 X-ray source and SPECS PHOIBOS 150 hemispherical electron analyzer in the Department of Physics at LSU. A clean TiO_2_ (110) surface with a small amount of oxygen vacancies (reduced surface) was prepared by a repeated cycles of Ne ion sputtering (Ne^+^, 1.5 kV, 10μA) and annealing (10 minutes at 650 °C) in the UHV vacuum. The presence of oxygen vacancies/defects on the clean TiO_2_ (110) surface leads to an excess of electrons in the surrounding areas of the defect sites. The formal oxidation state of the neighboring titanium cations is reduced from 4+ to 3+. X-ray photoelectron data (not shown here) undertaken at room temperature adsorption of phenol on a reduced TiO_2_ (110) surface demonstrated that Ti 3+/Ti 4+ ratio decreases after dosing the surface with phenol. To corroborate this finding, we employed VUV photoelectron spectroscopy experiment at our new 5m-TGM beamline at CAMD. The phenol dosage was facilitated by introducing sublimated vapor into the UHV systems through a leak valve holding a pyrex tube loaded with solid phenol.

**Figure 3:**
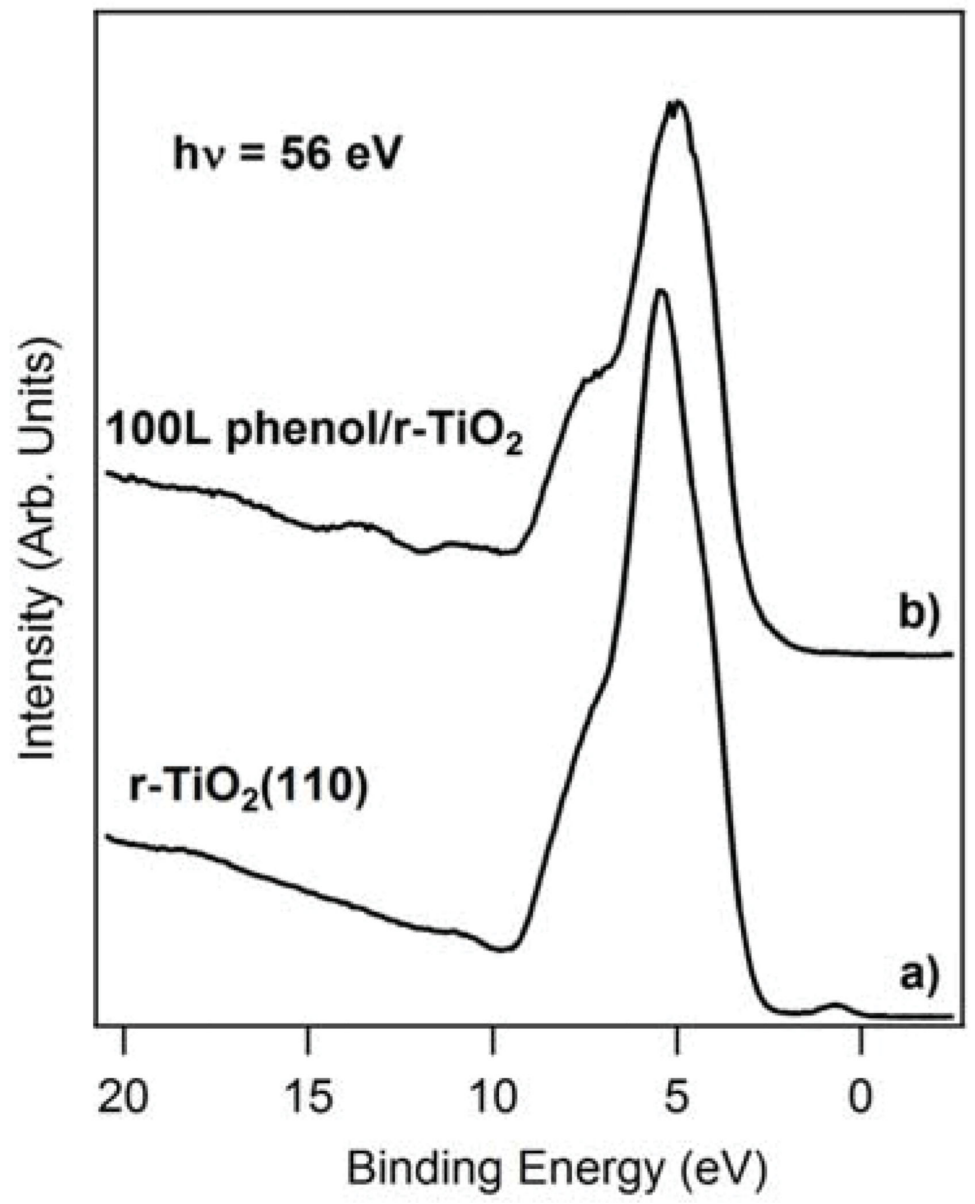
Photoelectron spectra of clean (a) and 100L phenol exposed (b) TiO_2_ (110).

Figure 3 shows the photoelectron spectra of both clean and 100 L phenol exposed TiO_2_ (110) surface. Both spectra were collected with photon energy of 56 eV, which is within the range of the Ti resonance energies (3p-4s transition) to enhance the intensity of Ti defect states. On the clean photoelectron spectrum (figure 3a), oxygen vacancies induce a defect state that appears at 1 eV binding energy. The features between 3-9 eV are mainly O-2p states hybridized with Ti-3d states. Upon phenol adsorption new electronic features appeared on the valence band at a binding energies of 2.4 eV, 10.7 eV, 13.5 eV and 16.7 eV as seen in the figure 3b. Very similar features were also observed for catechol adsorption on TiO_2_ (110) [5]. The structural changes of the O-2p band indicate phenol derived molecular orbitals existence in the binding energy range of 3-9 eV. The defect state appearing at 1 eV binding energy on the clean reduced TiO_2_ (110) has almost completely vanished after dosing 100 L phenol at RT. VUV photoelectron spectroscopy results indicate that phenol adsorbs on the surface of TiO_2_ and occupies the oxygen vacancies sites, which supports the X-ray photoelectron results we independently performed. It is also suggested that phenol adsorption on TiO_2_ (110) presumably oxidizes the metal ion (Ti). We are currently performing theoretical calculations to compare with the results obtained from X-ray and VUV photoelectron spectroscopy measurements.
